# Molecular profiling of 888 pediatric tumors informs future precision trials and data-sharing initiatives in pediatric cancer

**DOI:** 10.1038/s41467-024-49944-0

**Published:** 2024-07-11

**Authors:** Suzanne J. Forrest, Hersh Gupta, Abigail Ward, Yvonne Y. Li, Duong Doan, Alyaa Al-Ibraheemi, Sanda Alexandrescu, Pratiti Bandopadhayay, Suzanne Shusterman, Elizabeth A. Mullen, Natalie B. Collins, Susan N. Chi, Karen D. Wright, Priti Kumari, Tali Mazor, Keith L. Ligon, Priyanka Shivdasani, Monica Manam, Laura E. MacConaill, Evelina Ceca, Sidney N. Benich, Wendy B. London, Richard L. Schilsky, Suanna S. Bruinooge, Jaime M. Guidry Auvil, Ethan Cerami, Barrett J. Rollins, Matthew L. Meyerson, Neal I. Lindeman, Bruce E. Johnson, Andrew D. Cherniack, Alanna J. Church, Katherine A. Janeway

**Affiliations:** 1 Dana-Farber/Boston Children’s Cancer and Blood Disorders Center, Boston, MA USA; 2 Harvard Medical School, Boston, MA USA; 3 Dana-Farber Cancer Institute, Boston, MA USA; 4 Broad Institute of MIT and Harvard, Cambridge, MA USA; 5 Boston Children’s Hospital, Boston, MA USA; 6 Brigham and Women’s Hospital, Boston, MA USA; 7 American Society of Clinical Oncology, Alexandria, VA USA; 8 National Cancer Institute, Bethesda, MD USA; 9 Weill Cornell Medical College, New York, NY USA

**Keywords:** Paediatric cancer, Cancer genomics

## Abstract

To inform clinical trial design and real-world precision pediatric oncology practice, we classified diagnoses, assessed the landscape of mutations, and identified genomic variants matching trials in a large unselected institutional cohort of solid tumors patients sequenced at Dana-Farber / Boston Children’s Cancer and Blood Disorders Center. Tumors were sequenced with OncoPanel, a targeted next-generation DNA sequencing panel. Diagnoses were classified according to the International Classification of Diseases for Oncology (ICD-O-3.2). Over 6.5 years, 888 pediatric cancer patients with 95 distinct diagnoses had successful tumor sequencing. Overall, 33% (n = 289/888) of patients had at least 1 variant matching a precision oncology trial protocol, and 14% (41/289) were treated with molecularly targeted therapy. This study highlights opportunities to use genomic data from hospital-based sequencing performed either for research or clinical care to inform ongoing and future precision oncology clinical trials. Furthermore, the study results emphasize the importance of data sharing to define the genomic landscape and targeted treatment opportunities for the large group of rare pediatric cancers we encounter in clinical practice.

## Abstract (web summary)

Mutation profiling of pediatric cancers can help determine treatment options, however, large-scale datasets are rare. Here, the authors describe an institutional application of targeted sequencing to pediatric solid tumours, and identify potential therapeutic implications for identified mutations.

## Introduction

Over the last 50 years, there has been a profound prolongation in the survival of children and adolescents with cancer, primarily achieved through the intensification of chemotherapy, risk stratification, and multi-modal treatments^1,2^. Despite these advances, cancer remains the leading cause of death by disease among children in the United States, and many survivors of childhood cancers have significant long-term sequelae from their treatment^3–6^. Furthermore, progress has not been universal, and a number of specific cancer diagnoses have seen little improvement in outcomes and continue to have a disproportionate burden of treatment-related side effects. Specific groups with lagging improvement in outcomes include pediatric, adolescent, and young adult (AYA) brain tumors and sarcomas^1^.

One of the major drivers of improved outcomes and decreased toxicity of therapy is the application of precision oncology, carried out by cancer type-specific characterization of the genome paired with molecularly targeted therapy using investigational or approved agents^7,8^. In many cancers, genomic characterization has also facilitated the classification of biological subtypes associated with treatment response and resistance and the development of risk-stratified treatment protocols^8–10^. Pediatric brain and extracranial solid tumors are a group of ultra-rare malignancies occurring in pediatric and AYA patients^11^. The rarity of many pediatric brain and solid tumors is a barrier to generating clinical-genomic databases containing sufficient patients for meaningful genomic analyses to guide precision oncology and has slowed progress in these diseases.

At Dana-Farber / Boston Children’s Cancer and Blood Disorders Center, since 2013, all pediatric patients with cancer or suspected cancer have been eligible for enrollment in the Profile Cancer Research Study, an institutional sequencing study generating clinical grade targeted next-generation sequencing (NGS) reports which are returned to treating physicians and the medical record^12^. The study was universally offered to pediatric brain and solid tumor patients. The resulting data presents an opportunity to perform analyses of the genomic features of these rare and ultra-rare pediatric cancers, facilitating clinical trial design and real-world practice in precision oncology. In addition, these data can be contributed to data-sharing initiatives in pediatric cancer, including the National Cancer Institute’s (NCI) Childhood Cancer Data Initiative (CCDI)^13,14^.

## Results

### Patient and sample characteristics: sequenced cases have a long tail of ultra-rare diagnoses

**Fig. 1:**
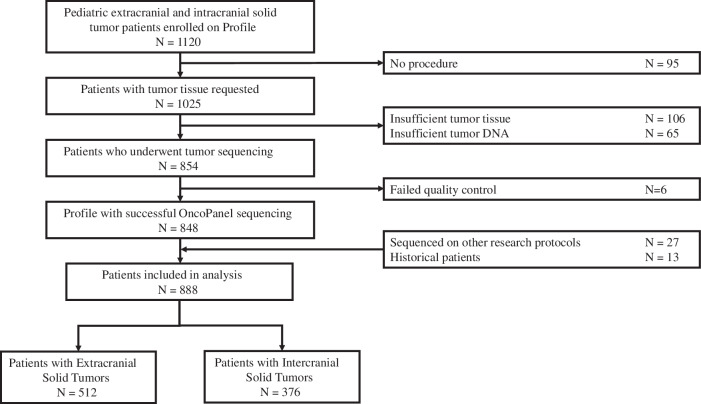
CONSORT diagram of analytic cohort. Flow chart illustrating derivation of the final study cohort.

Between September 2013 and March 2019, 1120 pediatric patients with intracranial (CNS) or extracranial solid tumors consented to and enrolled in the Profile Cancer Research Study. Targeted NGS of tumor samples was performed on tissue obtained at the time of a clinically indicated procedure. The OncoPanel assay, performed at the Center for Advanced Molecular Diagnostics (CAMD) at the Brigham and Women’s Hospital, was successful for 76% (848/1120) of enrolled participants. Sufficient tumor tissue from a previous clinical procedure was unavailable for 201 of the 1120 enrolled participants (18%). Seventy-one participants (6%) had insufficient or low-quality tumor DNA after extraction (Fig. 1).

OncoPanel data from an additional 27 patients enrolled at Dana-Farber in a similar, previously published study^15^, were also included in this analysis. Data from 13 patients with extracranial solid tumors sequenced with OncoPanel with a waiver of informed consent were also included in the analysis (Fig. 1).

The final analytic cohort included 888 pediatric patients with solid tumors with successful somatic OncoPanel sequencing. Within this analytic population, 512 (58%) had extracranial solid tumors and 376 (42%) had CNS tumors. Median age at cancer diagnosis was 7.66 years; 56% of the patients were male, and 65% were white. The tumor stage at diagnosis was localized for 60% of the extracranial solid tumors, while 29% had metastatic disease. Most samples, 92%, were from the primary tumor site, and 73% were obtained at initial diagnosis prior to treatment. Most of the samples were sequenced with OncoPanel versions 2 and 3, with 12.6% sequenced with version 1 (Table 1).

**Table 1:** Clinical Characteristics of Patients and Sequenced Samples

	Solid Tumors *N* = 512	CNS Tumors *N* = 376	All *N* = 888
Age at diagnosis (years), n (%)			
0–5	220 (43.0%)	156 (41.5%)	376 (42.3%)
6–10	81 (15.8%)	93 (24.7%)	174 (19.6%)
11–15	113 (22.1%)	74 (19.7%)	187 (21.1%)
16–20	66 (12.9%)	44 (11.7%)	110 (12.4%)
>20	18 (3.5%)	3 (0.8%)	21 (2.4%)
Unknown	14 (2.7%)	6 (1.6%)	20 (2.3%)
Sex, n (%)			
Male	288 (56.2%)	213 (56.6%)	501 (56.4%)
Female	224 (43.8%)	163 (43.4%)	387 (43.6%)
Race, n (%)			
White	331 (64.6%)	246 (65.4%)	577 (65.0%)
Black or African American	17 (3.3%)	10 (2.7%)	27 (3.0%)
Asian	23 (4.5%)	22 (5.9%)	45 (5.1%)
Native Hawaiian or Other Islander	1 (0.2%)	0 (0%)	1 (0.1%)
Some other race	45 (8.8%)	40 (10.6%)	85 (9.6%)
Multiple Race	4 (0.8%)	1 (0.3%)	5 (0.6%)
Unknown/Not specified	91 (17.8%)	57 (15.2%)	148 (16.7%)
Ethnicity, n (%)			
Hispanic or Latino	41 (8.0%)	17 (4.5%)	58 (6.5%)
Non-Hispanic or Latino	452 (88.3%)	358 (95.2%)	810 (91.2%)
Unknown	19 (3.7%)	1 (0.3%)	20 (2.3%)
Stage at diagnosis, n (%)			
Localized	307 (60.0%)	119 (31.6%)	426 (48.0%)
Regional	25 (4.9%)	0 (0%)	25 (2.8%)
Metastatic	149 (29.1%)	18 (4.8%)	167 (18.8%)
Unknown	18 (3.5%)	1 (0.3%)	19 (2.1%)
Not applicable	13 (2.5%)	238 (63.3%)	251 (28.3%)
Timing of sequenced sample, n (%)			
Initial diagnosis before treatment	365 (71.3%)	283 (75.3%)	648 (73.0%)
Local control at initial diagnosis	80 (15.7%)	15 (4.0%)	95 (10.7%)
Relapse/progression before treatment	38 (7.4%)	57 (15.2%)	95 (10.7%)
Relapse/progression local control	13 (2.5%)	11 (3.0%)	24 (2.7%)
Autopsy	1 (0.2%)	0 (0%)	1 (0.1%)
Unknown	15 (2.9%)	10 (2.7%)	25 (2.8%)
Biopsy/resection site, n (%)			
Primary tumor	443 (86.5%)	369 (98.1%)	812 (91.5%)
Metastatic site	66 (12.9%)	1 (0.3%)	67 (7.5%)
Unknown	3 (0.6%)	6 (1.6%)	9 (1.0%)
OncoPanel version, n (%)			
Version 1	68 (13.3%)	34 (9.4%)	112 (12.6%)
Version 2	221 (43.2%)	175 (46.5%)	396 (44.6%)
Version 3	223 (43.6%)	167 (44.4%)	390 (43.9%)

**Fig. 2:**
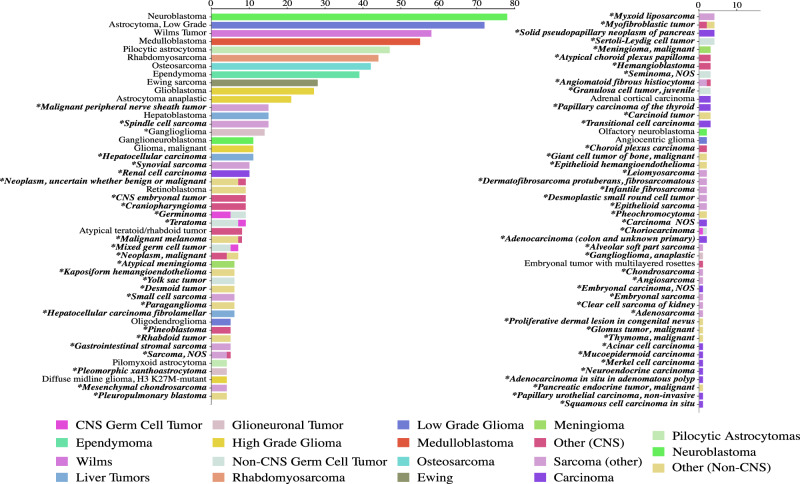
Longtail of patient diagnoses classified by ICD-O-3.2. The number of tumors sequenced with each pathologic diagnosis are shown. Diagnoses are color coded by disease sub-group. Diagnoses marked with * and in bold were not included in two prior pediatric pan-cancer sequencing studies^16,17^.

Patient diagnoses were confirmed and uniformly classified according to ICD-O-3.2 by a multidisciplinary team review of the pathology reports. 95 distinct histologic cancer diagnoses were represented in the study cohort. While 55% (451/888) of the patients in the cohort had one of ten common pediatric cancers, the remaining 45% of participants (398/888) had one of 85 distinct rare pediatric cancer diagnoses. The common pediatric cancer diagnoses were: neuroblastoma (*n* = 78), low-grade astrocytoma (*n* = 72), Wilms tumor (*n* = 58), medulloblastoma (*n* = 55), pilocytic astrocytoma (*n* = 47), rhabdomyosarcoma (*n* = 44), osteosarcoma (*n* = 42), ependymoma (*n* = 39), Ewing sarcoma (*n* = 28), and glioblastoma multiforme (*n* = 27) (Fig. 2). For the 85 distinct rare pediatric cancers, there were fewer than 25 patients per histologic diagnosis.

### Sequenced cases are representative of national pediatric cancer registries and contain diagnoses not present in prior pan-pediatric cancer sequencing analyses

**Fig. 3:**
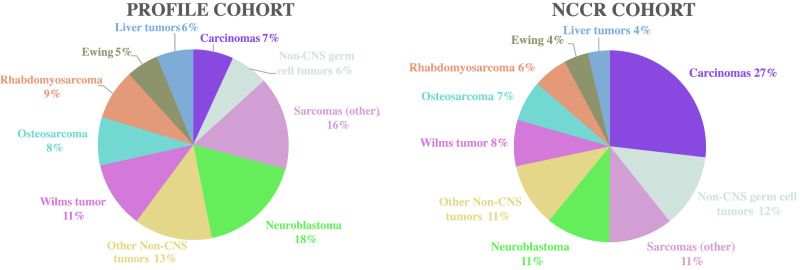
The distribution of pediatric extracranial solid tumor diagnoses represented in this study cohort compared to the NCCR Registries 2014-2018. The percentage of patients with each extracranial solid tumor diagnosis or diagnosis group is shown.

Proportionally, extracranial solid tumor diagnoses in this analytic cohort are similar to the National Cancer Institute (NCI) National Childhood Cancer Registry (NCCR) pediatric (ages < 20) population from 2014–2018^11^, with the exception of carcinomas which were underrepresented in our cohort (6.8%) compared to NCCR cohort (26.8%) (Fig. 3). This difference is likely driven by adolescent and young adult patients in the NCCR registry with thyroid carcinomas (14.5%), as these patients are often not referred to our pediatric oncology practice. There are two often cited landmark pediatric pan-cancer sequencing analyses^16,17^. All 10 common pediatric diagnoses are included in these pan-pediatric cancer analyses, while 75 of the 85 (88%) rare cancer diagnoses in our analytic population are not represented (Fig. 2).

### Pediatric and AYA cancers frequently harbor genomic variants matching targeted therapy basket trials but real-world precision oncology practice often involves off-label treatment

**Fig. 4:**
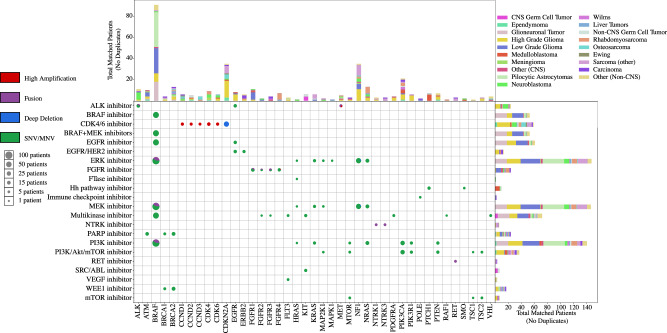
Patients with oncogenic tumor variants matching to a treatment arm of the NCI-COG Pediatric MATCH Trial, NCI-MATCH Trial, or ASCO TAPUR Study. Gene altered and corresponding matching targeted therapy type are shown. The size of each dot represents the number of patients with a matching variant in that gene, and the color of the dot represents the type of variant. Matched patients’ diagnosis grouping is shown by gene and type of targeted treatment.

To identify patients with tumor variants that would have constituted eligibility criteria for a clinical trial of a molecularly targeted therapy or for which there are clinically indicated agents, we used the actionable mutation of interest (aMOI) lists from three large precision medicine basket trials: the NCI-Children’s Oncology Group (COG) Pediatric MATCH (Molecular Analysis for Therapy Choice) trial^18^; the NCI-MATCH trial^19^; and the ASCO (American Society of Clinical Oncology) TAPUR (Targeted Agent and Profiling Utilization Registry) Study (Supplementary Data 1***)***^20^. Overall, 33% (*n* = 289/888) of patients had tumors with at least 1 oncogenic genomic alteration that matched to a targeted treatment arm of at least one of the three precision oncology basket trials (Fig. 4)^18–20^. The number of patients with tumors that had an actionable variant matching a treatment arm of the NCI-COG Pediatric MATCH Trial, the NCI-MATCH Trial, and the ASCO TAPUR Study were 238, 227, and 124, respectively. Seventy-five patients had a tumor variant matching an arm of all 3 trials. The genes that most frequently contained aMOIs were: *BRAF* (10%), *NF1* (4%), *CDKN2A* (4%), *PI3KCA* (2.4%), *NRAS/KRAS* (2.1%), *BRCA2* (1.5%), *ALK* (1.2%), and *FGFR1* (1.2%) (Fig. 4).

Many patients (*n* = 219) had tumors with variants that matched multiple treatment arms, and some patients (*n* = 64) had tumors with multiple matching genomic alterations. The proportion of patients with variants matching precision oncology treatment protocols differed by diagnosis. Glioneuronal tumors, high-grade gliomas, and pilocytic astrocytomas had the highest match rates at 89%, 70%, and 64%, respectively, driven by BRAF alterations (Supplementary Table 1). Ewing sarcoma and Wilms tumor had the lowest match rate with only 7% and 12%, respectively, of patients with those diagnoses having a tumor variant matching a targeted therapy treatment arm (Supplementary Table 1).

**Fig. 5:**
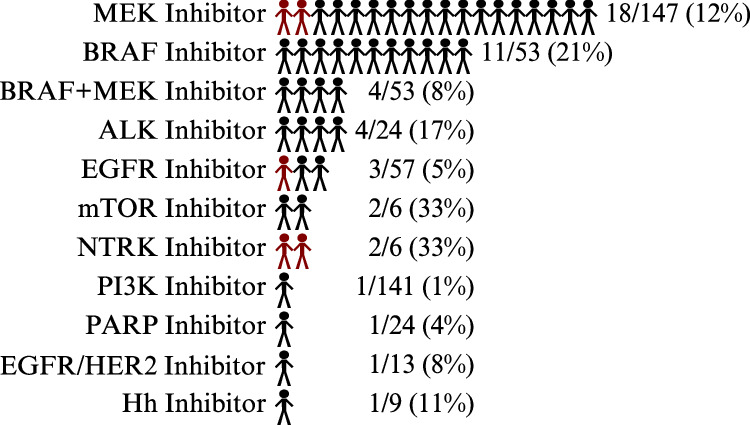
Patients who received molecularly targeted therapy to identified aMOIs. The number and proportion of patients who received matched therapy is shown by inhibitor type. The black color indicates receipt of matched therapy via single patient protocol or off-label, and the red color indicates receipt of matched therapy on a clinical trial.

We reviewed the prescription data and medical records of patients with tumors containing precision medicine basket trial aMOIs to determine whether they had received molecularly targeted therapy matching the identified aMOI. Of the 289 patients who had tumors with aMOIs, 41 (14%) received 48 matched molecularly targeted therapies (Fig. 5). Two patients received 2 matched therapies, and 2 patients received 3 matched therapies. Ten classes of molecularly targeted therapies were received, as shown in Fig. 5. Only 12% (5/41) of the patients who received a matched molecularly targeted therapy were enrolled in a clinical trial, with 88% (36/41) of patients receiving the matched therapy via single patient protocol or off-label (Fig. 5).

### Analysis of recurrent genomic alterations in rare, previously understudied pediatric cancers reveals unique opportunities for targeted therapy

We analyzed recurrent genomic alterations in this patient population separately for extracranial solid malignancies and CNS tumors. We then also assessed the oncogenic mutations uniquely enriched in the 75 rare extracranial solid tumors and CNS diagnoses that were not included in the two previously reported large pan-pediatric cancer analyses^16,17^. The most common recurrent oncogenic alterations occurring in > 5% of the extracranial solid tumor cases were *TP53* mutation/deletions (9%), *MYC/MYCN* amplifications (6%), and *CTNNB1* mutations (6%) (Supplementary Fig. 1). In the CNS tumors, the most common oncogenic alterations occurring in > 5% of the cases were *BRAF* rearrangements/mutations (21%), *TP53* mutations/deletions (12%) and *CDKN2A/B* deletions (6%) (Supplementary Fig. 2).

We observed the expected patterns of genomic events in specific diagnoses, including *TP53* rearrangements in osteosarcoma, *EWSR1* rearrangements in Ewing sarcoma, *ALK* mutations and *MYCN* amplification in neuroblastoma, *BRAF* fusions and *IDH1* mutations in low-grade glioma and *TP53* mutations, *CDKN2A/B* deletions and *H3F3A* mutations in high-grade gliomas. In contrast, activating *PIK3CA* gene alterations, present in 18 cases (2% of the entire cohort), were distributed across cancer diagnoses with 6 different extracranial solid tumors and 5 different CNS solid tumor diagnoses containing these alterations. Similarly, *ARID1A* inactivating mutations, present in 10 cases (1.1% of the entire cohort), were present in 8 different histologies (8 extracranial and 2 CNS) (Supplementary Table 2*,* Supplementary Fig. 1 and Supplementary Fig. 2).

**Fig. 6:**
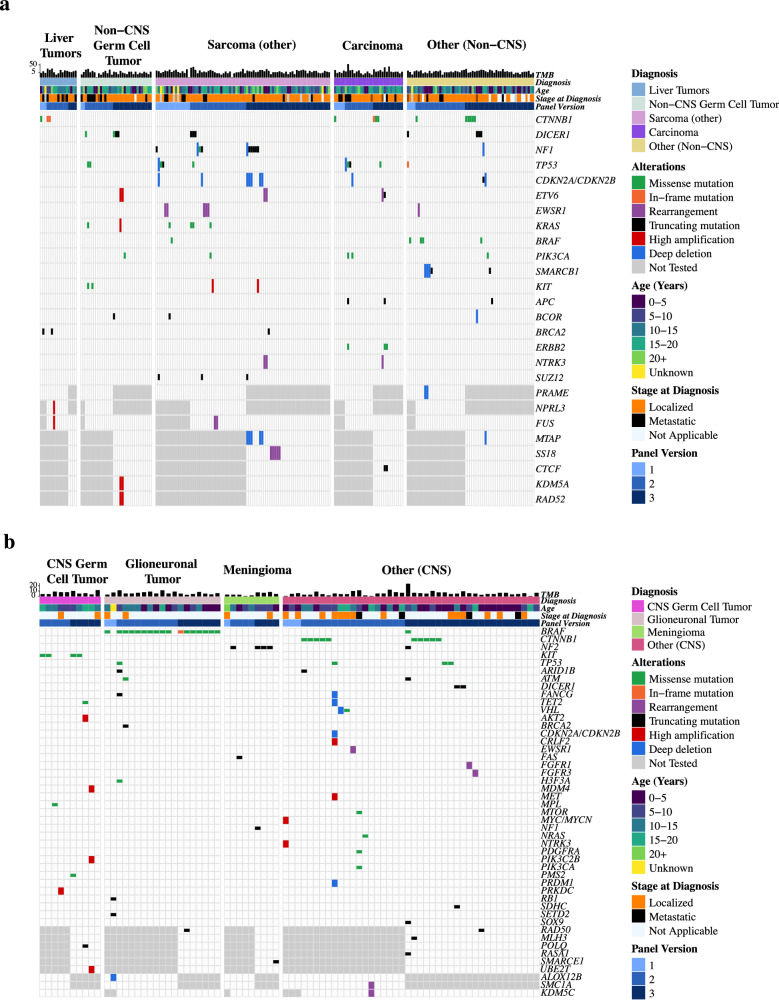
OncoPrint showing most common oncogenic alterations in the histologies not included in two prior pediatric pan-cancer sequencing studies. Alterations with > 1% frequency within the shown patients are displayed along with clinical features of each case for **a** Extracranial solid tumors (*n* = 235) and **b** CNS tumors (*n* = 75).

In the 235 extracranial solid tumors with histologies not included in prior pediatric pan-cancer analyses, the recurrently altered genes uniquely containing oncogenic alterations (compared to the common tumors) were *CTNNB1*, *DICER1*, and *NF1* (Fig. 6a). In the 78 CNS tumors with histologies not included in prior pediatric pan-cancer analyses, the recurrently altered genes uniquely containing oncogenic alterations (compared to the common tumors) were *CTNNB1*, *NF2* and *KIT* (Fig. 6b). Genomic alterations potentially targetable with precision therapeutics uniquely present in the rare CNS and extracranial solid tumors include *ERBB2* activating mutations in carcinomas, *KIT* activating mutations in CNS and non-CNS germ cell tumors, and *CTNNB1* inactivating mutations in carcinomas, liver tumors, desmoid tumors, and craniopharyngiomas (Fig. 6). In addition to these therapeutically actionable variants, *DICER1* alterations were present in 4% (13/313) of the rare diagnoses, with potential implications for both diagnosis and germline cancer predisposition (Fig. 6).

### Sequencing data linked to electronic medical records presents an opportunity for data sharing

The sequencing data for this study were shared with the NCI’s CCDI. Because these sequencing data retained a link to the patient’s Dana-Farber / Boston Children’s Cancer and Blood Disorders electronic medical record (EMR), there was the opportunity to annotate these genomic data more fully to provide a complete picture of the cancer diagnosis, treatment, and outcome. For selected osteosarcoma, Ewing sarcoma, Wilms tumor, and neuroblastoma patients included in the analytic population, we used the PRISSMM^TM^ data model^21^ and trained data abstractors to obtain these additional clinical data from the EMR. The EMR for 38, 29, 25, and 20 patients with Wilms tumor, osteosarcoma, Ewing sarcoma, and neuroblastoma, respectively, were abstracted into the PRISSMM^TM^ data model in RedCap (Research Electronic Data Capture)^22,23^. For these patients, the deeper clinical annotation includes Toronto Stage^24^ on 92 patients, an average of 4 pathology reports, and 21 imaging reports per patient, with a range of 1–29 reports per patient for pathology and 1–110 reports per patient for imaging. The average number of cancer treatment regimens captured in the data per patient is 3, with a range of 1–12. The median follow-up time for these patient data is 38 months with a range of 0-300 months.

## Discussion

Patients with CNS and extracranial solid tumors seen by pediatric oncology at Dana-Farber/Boston Children’s Cancer and Blood Disorders Center were offered the opportunity to participate in a cohort study of tumor profiling with the return of results. The study enabled us to generate, analyze, and share sequencing and clinical data for both common and rare pediatric malignancies. Initial efforts sequencing pediatric cancers, such as the Therapeutically Applicable Research to Generate Effective Treatments (TARGET) Childhood Cancer Program, understandably focused on more common pediatric cancers^25–28^. As a result, the initial pan-pediatric cancer mutational landscape analyses were biased towards more common pediatric solid tumors and cancer types of specific interest to the investigators. In contrast, studies like ours enrolling any pediatric cancer patient in a cohort study with the return of sequencing results generate data on a complete spectrum of pediatric solid tumors. As shown in Table 2, Genomes for Kids and Pediatric MATCH enrolled a similarly large number of distinct diagnoses^29,30^. Due to the prevalence of diagnoses in the long tail of diverse pediatric solid tumors, biologic insights for the ultra-rare cancers in this and similar studies will require data sharing to fully exploit the potential therapeutic opportunities. For example, a combined dataset would permit a better understanding of genomic data presented in this analysis, such as a more precise estimate of the frequency of targetable *PIK3CA* mutations in pediatric solid tumors and a better understanding of the diagnoses in which they occur. We have contributed the data from a subset of this analytic cohort to GENIE (Genomics Evidence Neoplasia Information Exchange) and from the entire cohort to the NCI’s Childhood Cancer Data Initiative (see “Methods”, Data Availability sections for more information).

**Table 2:** Characteristics of current study and 5 recently published clinical sequencing/matching protocols

	Current	Genomes for Kids	INFORM	MAPPYACTS	Pediatric-MATCH	Zero Childhood Cancer
Patients, n	888	253	926	624	1000	247
Age, y	0–25	0–25	0–40	0–38	1–21	0–31
Diagnosis Group, %						
CNS	42%	31%	27%	29%	25%	37%
HM	–	41%	9%	10%	3%	17%
ST	58%	27%	64%	61%	72%	46%
Disease Status, %						
Initial diagnosis	84%	85%	–	–	–	47%
R/R or high-risk	14%	15%	100%	100%	100%	51%
Histologies, n	95	81	17 + other	36	80	24 + other
Type of Sequencing	Panel	WES	WES	WES	Panel	WGS
		WGS	icWGS	RNAseq		RNAseq
		RNAseq	RNA seq Methylation	Panel		Methylation
Year Published		2021	2021	2022	2022	2020
PMID		34301788^29^	34373263^37^	35292802^32^	35353553^30^	33020650^55^

Shared genomic data with paired clinical data, including baseline disease features, treatment, and outcomes, will allow the scientific community to identify genomic features of disease that are biomarkers for poor outcomes or that predict treatment response or resistance mechanisms. As such, developing standards for categorizing and reporting clinical data from the EMR is an ongoing high priority. Given the number of rare pediatric solid tumors, diagnosis classification is an essential component of data standardization. Here, we show that classifying pediatric cancer diagnoses using the ICD-O standard ontology system is feasible, and we believe a prospective approach to diagnosis classification within the EMR would benefit research use of clinical data and help facilitate current and future data-sharing initiatives in pediatric solid tumors. In addition, we adapted the PRISSMM^TM^ model to several pediatric cancers and shared this data, with a code enabling linkage to genomic data, with the CCDI’s NCCR initiative.

Molecular tumor profiling tests are becoming a more common component of clinical care for pediatric patients with solid malignancies^15^. Observations from this study shed light on what clinicians can expect when sequencing pediatric patients with solid malignancies in the clinic. Similar to other studies, almost 20% of the patients enrolled in this study did not have tumor tissue available (8% had no procedure, 9% had insufficient tumor) for sequencing^31,32^. This is partly due to a high rate of second opinion visits in our pediatric brain and solid tumor programs. Other contributing factors include small biopsies for which leftover specimens were not available and past tissue processing practices in pathology, such as harsh decalcification of bone sarcomas^33^. The technical failure rate of 6% is consistent with failure rates previously reported by us and other groups, including the Pediatric MATCH trial^30^. We report a 33% rate of detecting genomic variants which would make patients potentially eligible for clinical trial arms of basket precision oncology trials, which is essentially the same as the rate reported (31.5%) for the Pediatric MATCH screening protocol^30^. Of note, the proportion of patients matching precision oncology treatment trials was the same for the Pediatric and Adult MATCH trials supporting current drug development policies of considering pediatric trials for molecularly targeted therapies^34,35^.

These results highlight the importance of obtaining molecular characterization of pediatric CNS and extracranial solid tumors. The extent to which molecular testing has been incorporated into the standard care of these patients varies by treatment setting, country, and diagnosis. For example, there are national research programs in the United States and several European countries offering sequencing, including, in many cases RNAseq, for either all pediatric cancers or specific diagnoses^36–38^. In the United States, there are national guidelines for NGS of adult cancers, which provides support for insurance reimbursement for molecular testing^39–41^. However, guidelines for pediatric tumors are more limited but beginning to be established for select diagnoses^42,43^. Continued efforts to address the role of molecular profiling for pediatric cancers will be important as these guidelines are developed. Diagnosis-specific guidelines will not fully address the role of sequencing in the ultra-rare histologies constituting 45% of the cases in this study, and an ultra-rare pediatric cancer or pan-pediatric cancer guideline or statement will likely be needed. In the interim, continued efforts to molecularly profile pediatric CNS and extracranial solid tumors, particularly ultra-rare and advanced cancers, for diagnostic, prognostic, and therapeutic purposes are critically important.

Only 14% of the 289 patients with an aMOI and 5% of the overall sequenced population received molecularly targeted therapy matched to an identified genomic variant. There are several possible reasons for the low proportion of patients with an aMOI treated with targeted therapy, which could be explored in future studies. Patients were most often enrolled at diagnosis and may have completed standard therapy without the need for further therapy. Furthermore, treatment data may be missing for a subset of patients seen for second opinions. Lastly, the era in which the sequencing was performed extends back to 2013, when fewer molecularly targeted treatments were available. Interestingly, molecularly targeted therapy was received outside of a clinical trial for 88% of patients, a similar finding to our recent report in relapsed and refractory extracranial solid tumors (72% of patients treated off trial)^44^. This finding suggests the need for future efforts to collect high-quality treatment and outcome data from EMRs in order to understand dosing, administration, efficacy, and toxicity data for molecularly targeted therapies used in pediatric solid tumor patients.

The major limitation of this study is that sequencing was from tumor only and utilized a targeted panel. As a result, it is challenging to perform mutational signature analysis. Furthermore, sequencing data may, in some cases, contain germline variants that could be inappropriately classified as somatic^45^. At Dana-Farber Cancer Institute, in collaboration with the Brigham and Women’s Hospital, we have now launched a paired tumor-germline targeted sequencing assay to address these issues and patients are currently eligible to enroll in a study to access this assay.

## Methods

### Study participants and tumor samples

This study complies with all relevant ethical regulations and was approved by the Dana-Farber Cancer Institute (DFCI) Institutional Review Board (IRB). All patients seen at Dana-Farber/Boston Children’s Hospital Cancer and Blood Disorders Center with a suspected or confirmed cancer were eligible to participate in the Profile Cancer Research Study starting in 2013^12^. There was no age limit, and patients were considered pediatric if they were seen by a pediatric provider. Between 2013 and 2019 all pediatric patients with a brain tumor or extracranial solid tumor were offered the opportunity to enroll in the study. Patients and their families who provided written informed consent underwent targeted NGS sequencing of tumor specimens collected for clinical purposes (eg. biopsy or resection) in a Clinical Laboratory Improvement Amendments (CLIA)-certified clinical laboratory. Sequencing results were returned to the physician and medical record. Pediatric patients consented to the Profile Study between September 2013 and March 2019 with a solid tumor and successful tumor sequencing were included in this analysis. More than four years have passed since the last patient was enrolled, so adequate time has now passed for many of the patients to utilize the genomic information for potential treatment based on the genomic findings. Pediatric patients enrolled on Profile with a hematologic malignancy, or a benign tumor were excluded. Tumor samples were requested from the pathology department following patient consent after the standard pathology evaluation was complete. Tumor sample acquisition procedures were not altered for these studies, and these clinical samples were most often leftover FFPE (formalin fixed paraffin embedded) specimens in the pathology department. Several additional patients who underwent targeted NGS sequencing with OncoPanel on a similar multi-institution sequencing study^15^ and a small number of tumor samples sequenced under a waiver of consent were also included if they had a spindle or round cell sarcoma. If patients had multiple tumor samples sequenced at different time points, then only one was used for analysis. Tumor sample acquired at the time of initial diagnosis was used when available, and if not available, the sample with better quality was used based on pre-determined criteria (pre-treatment samples at the earliest available relapse/recurrence were prioritized over post-treatment specimens).

### Clinical data collection

The medical records of all the patients with successful OncoPanel sequencing were reviewed to determine clinical and demographic characteristics, including sex, race (self-reported), ethnicity (self-reported), pathologic diagnosis, age at diagnosis, and disease stage at diagnosis. Characteristics for the specimen that underwent sequencing were also extracted, including timing of sample acquisition including relationship to treatment, and site of the tumor (primary site vs. metastatic). The pathologic diagnosis was classified according to the International Classification of Diseases for Oncology, version 3.2 (ICD-O-3.2)^46^ following an expert committee review of the pathology report (S.J.F., A.A., S.A., K.L.L., P.B., A.J.C., K.A.J.) for each sequenced tumor sample. The expert committee included pediatric oncologists and neuro-oncologists, pediatric pathologists with neuropathology, and sarcoma expertise. Diagnoses were classified as extracranial or intracranial solid tumors, and further sub-classified into disease groupings per Supplementary Data 2.

For selected osteosarcoma, Ewing sarcoma, Wilms tumor, and neuroblastoma patients included in the analytic population, we used the PRISSMM^TM^ data model^21^ and trained data abstractors to obtain additional clinical data from the EMR. The PRISSMM^TM^ model developed for adult solid tumors was adapted to these four pediatric malignancies by modifying cancer-type specific fields and adding specific prognostically important biomarkers. In addition, we obtained baseline imaging data from scan reports to enable derivation of Toronto Stage^24,47^. A curation guide was prepared for trained individuals to follow when abstracting data from the EMR and quality control was performed by dual abstraction for approximately 12% of cases. Clinical data were collected in RedCap^22,23^.

### Assessment of cohort generalizability

To determine the extent to which this cohort represents the larger pediatric solid and brain tumor patient population, data from the Cancer in North America (CiNA) North American Association of Central Cancer Registries (NAACCR) 1995–2018 and the NCI’s Surveillance, Epidemiology and End Results (SEER) Registries), submitted December 2020) were analyzed^11^. Registries include: California, Connecticut, Florida, Georgia, Hawaii, Idaho, Illinois, Iowa, Kentucky, Louisiana, Massachusetts, New Jersey, New Mexico, New York, Ohio, Pennsylvania, Seattle (Puget Sound), Tennessee, Texas, Utah, and Wisconsin. These 23 NCCR registries represent 66% of all U.S. children, adolescents, and young adults ages 0–39 based on 2018 U.S. Populations.

### Sequencing and data analysis

Tumors were sequenced using the targeted NGS OncoPanel platform as previously described^48–50^. Sequencing was performed at the Center for Advanced Molecular Diagnostics (CAMD), a CLIA-certified clinical laboratory in the Department of Pathology at Brigham and Women’s Hospital in Boston, Massachusetts. OncoPanel is a validated, targeted NGS panel of up to 447 cancer genes for the detection of single-nucleotide variants (SNV), insertions and deletions, and copy number alterations (CNA), as well as selected intronic regions for up to 60 genes for the detection of structural variants (SV). Samples were sequenced with multiple versions of OncoPanel (versions 1, 2, and 3) as gene coverage has expanded over time (gene coverage for each version utilized is provided in Supplementary Data 3). The variant allele fraction (VAF) cutoff used for OncoPanel reporting was 5%. However, lower VAF variants (minimum of 2.5%) were also included if assessed by the reporting molecular pathologist to be present with high confidence. A molecular pathology report was returned to treating providers at the time of sequencing.

For analyses of genomic variants, variant call files generated at the time of reporting were utilized. Additional filtering was applied to the existing pipeline output removing mutations found in either the ClinVar (v. 07_04_2019 release)^51^ or gnomAD v2.1^52^ databases. Tumor mutational burden was calculated by dividing the total remaining number of SNVs or small insertions and deletions (indels) by the total panel size for each version. SNVs and indels were classified as oncogenic if they were labeled as “Oncogenic”, “Likely Oncogenic”, or “Predicted Oncogenic” per the Memorial Sloan Kettering Precision Oncology Knowledge Base v3.4 (OncoKB)^53^. In addition, limited in-house curation was performed (YL, HG, SJF). Specifically, the following variants were further assessed for oncogenicity: (1) loss-of-function (LoF) mutations in tumor suppressor genes (TSG); (2) SNVs and Indels in genes on actionable mutation lists classified as variants of uncertain significance (VUS) and; (3) all fusions involving genes on the actionable mutation lists. OncoPrints were created using the ComplexHeatmap (v. 2.4.3) package^54^.

### Genomic associations with molecularly targeted therapy

Genomic alterations were analyzed and matched to the actionable mutation lists (aMOI) of three precision oncology medicine basket clinical trials investigating targeted therapy directed by tumor profiling: NCI-COG Pediatric MATCH Screening Trial (NCT03155620)^18^, NCI-MATCH Screening Trial (NCT02465060)^19^, and ASCO TAPUR Study, Version 3 (NCT02693535)^20^. Specific genomic alterations in the participant tumor samples were considered as matches using the following rules: (1) Precise match on either MATCH trial aMOI list (same CNA, SNV, Indel, or LoF mutation for tumor suppressors) taking into account resistance mutations; or (2) Same gene and variant type (eg. activating fusion, amplification or oncogenic SNV/Indel in an oncogene and LoF mutation or deletion in a TSG).

For patients with a tumor variant matching a basket trial treatment arm, the medical record was reviewed to determine whether the patient received a molecularly targeted therapy in the same drug class as the basket trial treatment arm. For patients who received molecularly targeted therapy, the mechanism of obtaining treatment (on a clinical trial, via single-patient research protocol, or prescribed off-study) was assessed.

### Statistics and reproducibility

No statistical method was used to predetermine sample size. No data were excluded from the analyses. The experiments were not randomized. The Investigators were not blinded to allocation during experiments and outcome assessment.

### Reporting summary

Further information on research design is available in the Nature Portfolio Reporting Summary linked to this article.

### Supplementary information

Supplementary Information

Peer Review File

Reporting Summary

Description of Additional Supplementary Files

Supplementary Data 1

Supplementary Data 2

Supplementary Data 3

### Source data

Source Data

## Data Availability

The genomic and clinical datasets generated and analyzed in this study were submitted to the National Cancer Institute’s Childhood Cancer Data Initiative (CCDI), and are available in the database of Genotypes and Phenotypes (dbGaP): Study Accession phs002677.v1.p1 [https://www.ncbi.nlm.nih.gov/projects/gap/cgi-bin/study.cgi?study_id=phs002677.v1.p1]. These data are available under restricted access due to individual privacy concerns, and requests are managed by NCI’s Data Access Committee. There are no restrictions on how long data will be made available. Full details of data access are available on the dpGAP webpage, but all additional queries may be sent to NCIDAC@mail.nih.gov. The comprehensive PRISSMMTM clinical data were shared with the Massachusetts State Cancer Registry, which is making it accessible to the National Childhood Cancer Registry (NCCR) and the CCDI. Annotation databases included public resources such as OncoKb, ClinVar, and gnomAD databases. Source data are provided with this paper.

## References

[1] Siegel, D. A. et al. Pediatric cancer mortality and survival in the United States, 2001-2016. *Cancer* **126**, 4379–4389 (2020). 32725630 10.1002/cncr.33080 PMC9539939

[2] Smith, M. A., Altekruse, S. F., Adamson, P. C., Reaman, G. H. & Seibel, N. L. Declining childhood and adolescent cancer mortality. *Cancer* **120**, 2497–2506 (2014). 24853691 10.1002/cncr.28748 PMC4136455

[3] Cunningham, R. M., Walton, M. A. & Carter, P. M. The major causes of death in children and adolescents in the United States. *N. Engl. J. Med.* **379**, 2468–2475 (2018). 30575483 10.1056/NEJMsr1804754 PMC6637963

[4] Bhakta, N. et al. The cumulative burden of surviving childhood cancer: an initial report from the St Jude Lifetime Cohort Study (SJLIFE). *Lancet* **390**, 2569–2582 (2017). 28890157 10.1016/S0140-6736(17)31610-0 PMC5798235

[5] Siegel, R. L., Miller, K. D., Fuchs, H. E. & Jemal, A. Cancer Statistics, 2021. *CA Cancer J. Clin.* **71**, 7–33 (2021). 33433946 10.3322/caac.21654

[6] Suh, E. et al. Late mortality and chronic health conditions in long-term survivors of early-adolescent and young adult cancers: a retrospective cohort analysis from the Childhood Cancer Survivor Study. *Lancet Oncol.* **21**, 421–435 (2020). 32066543 10.1016/S1470-2045(19)30800-9 PMC7392388

[7] Tran, T. H., Shah, A. T. & Loh, M. L. Precision medicine in pediatric oncology: translating genomic discoveries into optimized therapies. *Clin. Cancer Res.* **23**, 5329–5338 (2017). 28600472 10.1158/1078-0432.CCR-16-0115

[8] Pui, C. H. et al. Childhood acute lymphoblastic leukemia: progress through collaboration. *J. Clin. Oncol.* **33**, 2938–2948 (2015). 26304874 10.1200/JCO.2014.59.1636 PMC4567699

[9] Liang, W. H. et al. Tailoring therapy for children with neuroblastoma on the basis of risk group classification: past, present, and future. *JCO Clin. Cancer Inf.* **4**, 895–905 (2020). 10.1200/CCI.20.00074 PMC7608590 33058692

[10] Gajjar, A. et al. Outcomes by clinical and molecular features in children with medulloblastoma treated with risk-adapted therapy: results of an international phase III trial (SJMB03). *J. Clin. Oncol.* **39**, 822–835 (2021). 33405951 10.1200/JCO.20.01372 PMC10166353

[11] NCCR*Explorer: An interactive website for NCCR cancer statistics [Internet]. National Cancer Institute; 2021 Nov 8. [updated: 2021 Nov 8; cited 2022 Nov 21]. Available from: https://nccrexplorer.ccdi.cancer.gov.

[12] Sholl, L. M. et al. Institutional implementation of clinical tumor profiling on an unselected cancer population. *JCI Insight* **1**, e87062 (2016). 27882345 10.1172/jci.insight.87062 PMC5111542

[13] Flores-Toro, J. A. et al. The childhood cancer data initiative: using the power of data to learn from and improve outcomes for every child and young adult with pediatric cancer. *J. Clin. Oncol.* **41**, 10.1200/JCO.22.02208 (2023). 10.1200/JCO.22.02208 PMC10461939 37267580

[14] Plana, A. et al. Pediatric cancer data commons: federating and democratizing data for childhood cancer research. *JCO Clin. Cancer Inf.* **5**, 1034–1043 (2021). 10.1200/CCI.21.00075 34662145

[15] Harris, M. H. et al. Multicenter feasibility study of tumor molecular profiling to inform therapeutic decisions in advanced pediatric solid tumors: The individualized cancer therapy (iCat) study. *JAMA Oncol.* **2**, 608–615 (2016). 26822149 10.1001/jamaoncol.2015.5689

[16] Ma, X. et al. Pan-cancer genome and transcriptome analyses of 1,699 paediatric leukaemias and solid tumours. *Nature* **555**, 371–376 (2018). 29489755 10.1038/nature25795 PMC5854542

[17] Grobner, S. N. et al. The landscape of genomic alterations across childhood cancers. *Nature* **555**, 321–327 (2018). 29489754 10.1038/nature25480

[18] Allen, C. E. et al. Target and agent prioritization for the children’s oncology group-national cancer institute pediatric MATCH trial. *J. Natl. Cancer Inst.* **109,**10.1093/jnci/djw274 (2017). 10.1093/jnci/djw274 PMC5963793 28376230

[19] Murciano-Goroff, Y. R., Drilon, A. & Stadler, Z. K. The NCI-MATCH: A national, collaborative precision oncology trial for diverse tumor histologies. *Cancer Cell* **39**, 22–24 (2021). 33434511 10.1016/j.ccell.2020.12.021 PMC10640715

[20] Mangat, P. K. et al. Rationale and design of the targeted agent and profiling utilization registry (TAPUR) study. *JCO Precis. Oncol.* **2,**10.1200/PO.18.00122 (2018). 10.1200/PO.18.00122 PMC6312096 30603737

[21] Choudhury, N. J. et al. The GENIE BPC NSCLC cohort: a real-world repository integrating standardized clinical and genomic data for 1,846 patients with non-small cell lung cancer. *Clin. Cancer Res*. **29**, 3418–3428 (2023). 10.1158/1078-0432.CCR-23-0580 PMC10472103 37223888

[22] Harris, P. A. et al. The REDCap consortium: Building an international community of software platform partners. *J. Biomed. Inf.* **95**, 103208 (2019). 10.1016/j.jbi.2019.103208 PMC7254481 31078660

[23] Harris, P. A. et al. Research electronic data capture (REDCap)–a metadata-driven methodology and workflow process for providing translational research informatics support. *J. Biomed. Inf.* **42**, 377–381 (2009). 10.1016/j.jbi.2008.08.010 PMC2700030 18929686

[24] Gupta, S. et al. Paediatric cancer stage in population-based cancer registries: the Toronto consensus principles and guidelines. *Lancet Oncol.* **17**, e163–e172 (2016). 27300676 10.1016/S1470-2045(15)00539-2

[25] Pugh, T. J. et al. The genetic landscape of high-risk neuroblastoma. *Nat. Genet.* **45**, 279–284 (2013). 23334666 10.1038/ng.2529 PMC3682833

[26] Gadd, S. et al. A children’s oncology group and TARGET initiative exploring the genetic landscape of Wilms tumor. *Nat. Genet.* **49**, 1487–1494 (2017). 28825729 10.1038/ng.3940 PMC5712232

[27] Bolouri, H. et al. The molecular landscape of pediatric acute myeloid leukemia reveals recurrent structural alterations and age-specific mutational interactions. *Nat. Med.* **24**, 103–112 (2018). 29227476 10.1038/nm.4439 PMC5907936

[28] Brady, S. W. et al. The genomic landscape of pediatric acute lymphoblastic leukemia. *Nat. Genet.* **54**, 1376–1389 (2022). 36050548 10.1038/s41588-022-01159-z PMC9700506

[29] Newman, S. et al. Genomes for kids: the scope of pathogenic mutations in pediatric cancer revealed by comprehensive DNA and RNA sequencing. *Cancer Discov.* **11**, 3008–3027 (2021). 34301788 10.1158/2159-8290.CD-20-1631 PMC8783930

[30] Parsons, D. W. et al. Actionable tumor alterations and treatment protocol enrollment of pediatric and young adult patients with refractory cancers in the national cancer institute-children’s oncology group pediatric MATCH trial. *J. Clin. Oncol.* **40**, 2224–2234 (2022). 35353553 10.1200/JCO.21.02838 PMC9273376

[31] Parsons, D. W. et al. Diagnostic yield of clinical tumor and germline whole-exome sequencing for children with solid tumors. *JAMA Oncol.* **2**, 616–624 (2016). 26822237 10.1001/jamaoncol.2015.5699 PMC5471125

[32] Berlanga, P. et al. The european MAPPYACTS trial: precision medicine program in pediatric and adolescent patients with recurrent malignancies. *Cancer Discov.* **12**, 1266–1281 (2022). 35292802 10.1158/2159-8290.CD-21-1136 PMC9394403

[33] Pinches, R. S. et al. Making the most of small samples: Optimization of tissue allocation of pediatric solid tumors for clinical and research use. *Pediatr. Blood Cancer* **67**, e28326 (2020). 32667141 10.1002/pbc.28326

[34] RACE Act poised to advance pediatric cancer research. *Cancer Discov.* **10**, 1434–434 (2020). 10.1158/2159-8290.CD-NB2020-081 32826229

[35] Pearson, A. D. et al. Implementation of mechanism of action biology-driven early drug development for children with cancer. *Eur. J. Cancer* **62**, 124–131 (2016). 27258969 10.1016/j.ejca.2016.04.001

[36] O’Rourke, K. NCI launches the molecular characterization initiative for pediatric tumors. *Cancer* **128**, 3012 (2022). 35860918 10.1002/cncr.34381

[37] van Tilburg, C. M. et al. The pediatric precision oncology INFORM registry: clinical outcome and benefit for patients with very high-evidence targets. *Cancer Discov.* **11**, 2764–2779 (2021). 34373263 10.1158/2159-8290.CD-21-0094 PMC9414287

[38] Trotman, J. et al. The NHS England 100,000 genomes project: feasibility and utility of centralised genome sequencing for children with cancer. *Br. J. Cancer* **127**, 137–144 (2022). 35449451 10.1038/s41416-022-01788-5 PMC9276782

[39] Ettinger, D. S. et al. Non-small cell lung cancer, version 3.2022, NCCN clinical practice guidelines in oncology. *J. Natl. Compr. Canc. Netw.* **20**, 497–530 (2022). 35545176 10.6004/jnccn.2022.0025

[40] *FDA Fact Sheet: CDRH’S Approach to Tumor Profiling Next Generation Sequencing Tests* (US Food & Drug Administration, accessed December 22). https://www.fda.gov/media/109050/download (2023).

[41] CMS to cover NGS companion diagnostics. *Cancer Discov* **8**, 522 (2018). 10.1158/2159-8290.CD-NB2018-039 29615401

[42] Gajjar, A. et al. Pediatric central nervous system cancers, version 2.2023, NCCN clinical practice guidelines in oncology. *J. Natl. Compr. Canc. Netw.* **20**, 1339–1362 (2022). 36509072 10.6004/jnccn.2022.0062

[43] Balis, F. et al. Wilms tumor (Nephroblastoma), version 2.2021, NCCN clinical practice guidelines in oncology. *J. Natl. Compr. Canc Netw.* **19**, 945–977 (2021). 34416707 10.6004/jnccn.2021.0037

[44] Church, A. J. et al. Molecular profiling identifies targeted therapy opportunities in pediatric solid cancer. *Nat. Med*. **28**, 1581–1589 (2022). 10.1038/s41591-022-01856-6 PMC10953704 35739269

[45] Schienda, J. et al. Germline sequencing improves tumor-only sequencing interpretation in a precision genomic study of patients with pediatric solid tumor. *JCO Precis. Oncol.* **5**, 10.1200/PO.21.00281 (2021). 10.1200/PO.21.00281 PMC8710335 34964003

[46] Steliarova-Foucher, E., Stiller, C., Lacour, B. & Kaatsch, P. International classification of childhood cancer, third edition. *Cancer* **103**, 1457–1467 (2005). 15712273 10.1002/cncr.20910

[47] Gupta, S. et al. Development of paediatric non-stage prognosticator guidelines for population-based cancer registries and updates to the 2014 toronto paediatric cancer stage guidelines. *Lancet Oncol.* **21**, e444–e451 (2020). 32888473 10.1016/S1470-2045(20)30320-X

[48] Garcia, E. P. et al. Validation of oncoPanel: A targeted next-generation sequencing assay for the detection of somatic variants in cancer. *Arch. Pathol. Lab. Med.* **141**, 751–758 (2017). 28557599 10.5858/arpa.2016-0527-OA

[49] Abo, R. P. et al. BreaKmer: detection of structural variation in targeted massively parallel sequencing data using kmers. *Nucleic Acids Res.* **43**, e19 (2015). 25428359 10.1093/nar/gku1211 PMC4330340

[50] Nowak, J. A. et al. Detection of mismatch repair deficiency and microsatellite instability in colorectal adenocarcinoma by targeted next-generation sequencing. *J. Mol. Diagn.* **19**, 84–91 (2017). 27863258 10.1016/j.jmoldx.2016.07.010 PMC5225299

[51] Landrum, M. J. et al. ClinVar: improving access to variant interpretations and supporting evidence. *Nucleic Acids Res.* **46**, D1062–D1067 (2018). 29165669 10.1093/nar/gkx1153 PMC5753237

[52] Karczewski, K. J. et al. The mutational constraint spectrum quantified from variation in 141,456 humans. *Nature* **581**, 434–443 (2020). 32461654 10.1038/s41586-020-2308-7 PMC7334197

[53] Chakravarty, D. et al. OncoKB: A precision oncology knowledge base. *JCO Precis. Oncol.* **1**, 10.1200/PO.17.00011 (2017). 10.1200/PO.17.00011 PMC5586540 28890946

[54] Gu, Z., Eils, R. & Schlesner, M. Complex heatmaps reveal patterns and correlations in multidimensional genomic data. *Bioinformatics.* **32**, 2847–2849 (2016). 27207943 10.1093/bioinformatics/btw313

[55] Wong, M. et al. Whole genome, transcriptome and methylome profiling enhances actionable target discovery in high-risk pediatric cancer. *Nat. Med.* **26**, 1742–1753 (2020). 33020650 10.1038/s41591-020-1072-4

