# Author Correction: Molecular profiling of 888 pediatric tumors informs future precision trials and data-sharing initiatives in pediatric cancer

**DOI:** 10.1038/s41467-024-51184-1

**Published:** 2024-08-22

**Authors:** Suzanne J. Forrest, Hersh Gupta, Abigail Ward, Yvonne Y. Li, Duong Doan, Alyaa Al-Ibraheemi, Sanda Alexandrescu, Pratiti Bandopadhayay, Suzanne Shusterman, Elizabeth A. Mullen, Natalie B. Collins, Susan N. Chi, Karen D. Wright, Priti Kumari, Tali Mazor, Keith L. Ligon, Priyanka Shivdasani, Monica Manam, Laura E. MacConaill, Evelina Ceca, Sidney N. Benich, Wendy B. London, Richard L. Schilsky, Suanna S. Bruinooge, Jaime M. Guidry Auvil, Ethan Cerami, Barrett J. Rollins, Matthew L. Meyerson, Neal I. Lindeman, Bruce E. Johnson, Andrew D. Cherniack, Alanna J. Church, Katherine A. Janeway

**Affiliations:** 1 Dana-Farber/Boston Children’s Cancer and Blood Disorders Center, Boston, MA USA; 2 Harvard Medical School, Boston, MA USA; 3 Dana-Farber Cancer Institute, Boston, MA USA; 4 Broad Institute of MIT and Harvard, Cambridge, MA USA; 5 Boston Children’s Hospital, Boston, MA USA; 6 Brigham and Women’s Hospital, Boston, MA USA; 7 American Society of Clinical Oncology, Alexandria, VA USA; 8 National Cancer Institute, Bethesda, MD USA; 9 Weill Cornell Medical College, New York, NY USA

**Keywords:** Paediatric cancer, Cancer genomics

Correction to: *Nature Communications* 10.1038/s41467-024-49944-0, published online 11 July 2024

In this article the author’s name Andrew D. Cherniack was incorrectly written as Andrew D. Cherniak. The original article has been corrected.

