# Supplementary material for: Molecular profiling of 888 pediatric tumors informs future precision trials and data-sharing initiatives in pediatric cancer
Source: Nat Commun. 2024 Jul 11;15:5837. doi: 10.1038/s41467-024-49944-0 (PMC11239876; doi:10.1038/s41467-024-49944-0)

Supplementary Information for

Molecular Profiling of 888 Pediatric Tumors Informs Future Precision Trials and Data Sharing Initiatives in Pediatric Cancer

*Suzanne J. Forrest[1,2], Hersh Gupta[3,4], Abigail Ward[1], Yvonne Y. Li[3,4], Duong Doan[1], Alyaa Al Ibraheemi[2,5], Sanda Alexandrescu[2,5], Pratiti Bandopadhayay[1,2], Suzanne Shusterman[1,2], Elizabeth A. Mullen[1,2], Natalie B. Collins[1,2], Susan N. Chi[1,2], Karen D. Wright[1,2], Priti Kumari[3], Tali Mazor[3], Keith L. Ligon[2,3,5,6], Priyanka Shivdasani[6], Monica Manam[5], Laura E. MacConaill[6], Evelina Ceca[1], Sidney N. Benich[1], Wendy B. London[1], Richard L. Schilsky[7], Suanna S. Bruinooge[7], Jaime Guidry Auvil[8], Ethan Cerami[3], Barrett J. Rollins[2,3,6], Matthew L. Meyerson[2,3,4], Neal I. Lindeman[9], Bruce E. Johnson[2,3,6], Andrew D. Cherniak[3,4], Alanna J. Church[2,5], *Katherine A. Janeway[1,2]
*Corresponding authors

[1]Dana-Farber/Boston Children's Cancer and Blood Disorders Center, Boston, MA, USA
[2]Harvard Medical School, Boston, MA, USA
[3]Dana-Farber Cancer Institute, Boston, MA, USA
[4]Broad Institute of MIT and Harvard, Cambridge, MA, USA
[5]Boston Children's Hospital, Boston, MA, USA
[6]Brigham and Women's Hospital, Boston, MA, USA
[7]American Society of Clinical Oncology, Alexandria, VA, USA
[8]National Cancer Institute, Bethesda, MD, USA
[9]Weill Cornell Medical College, New York, NY, USA

Table of Consents

Supplementary Tables……………………………………………………………………………….……….3
Supplementary Table 1: Frequency of Targeted Therapy Matching Variant by Diagnosis Sub-Group.....3
Supplementary Table 2: Activating PIK3CA Gene Alterations and ARID1A Inactivating Alterations Present in Cohort………………………………………………………………………………………….4

Supplementary Figures……………………………………………………………………….………...…….5
Supplementary Figure 1: OncoPrint showing most common oncogenic alterations in the Extracranial solid tumors (n=512) across the entire analytic cohort ……………………………………...……….…...5
Supplementary Figure 2: OncoPrint showing most common oncogenic alterations in the CNS tumors (n=376) across the entire analytic cohort……………………………………………………………….…..6

Supplementary Table 1. Frequency of Targeted Therapy Matching Variant by Diagnosis Sub-Group

	Total # of Pts in Group	Matched Pts in Group	Frequency
**Glioneuronal Tumor**	19	17	89.47%
**High Grade Glioma**	63	44	69.84%
**Pilocytic Astrocytomas**	51	33	64.71%
**Low Grade Glioma**	79	41	51.90%
**Rhabdomyosarcoma**	44	18	40.91%
**CNS Germ Cell Tumor**	10	4	40.00%
**Carcinoma**	35	11	31.43%
**Sarcoma (other)**	81	22	27.16%
**Medulloblastoma**	55	14	25.45%
**Neuroblastoma**	91	22	24.18%
**Osteosarcoma**	42	10	23.81%
**Other (Non-CNS)**	68	16	23.53%
**Meningioma**	9	2	22.22%
**Liver Tumors**	32	6	18.75%
**Non-CNS Germ Cell Tumor**	33	6	18.18%
**Other (CNS)**	51	8	15.69%
**Ependymoma**	39	6	15.38%
**Wilms**	58	7	12.07%
**Ewing**	28	2	7.14%

Supplementary Table 2. Activating PIK3CA Gene Alterations and ARID1A Inactivating Alterations Present in Cohort

Gene	Protein Change	DNA Change	Diagnosis Group	Diagnosis Name
ARID1A	p.Q2037*	c.6109C>T	Carcinoma	Transitional cell carcinoma
ARID1A	p.R1742Sfs*21	c.5226_5249delAACGCTACTGGATCCTGGGAGGTTinsTC	Carcinoma	Adenocarcinoma, metastatic
ARID1A	p.Q543*	c.1627C>T	Ewing	Ewing sarcoma
ARID1A	p.Q1552*, p.W1670*	c.4654C>T, c.5010G>A	High Grade Glioma	Glioblastoma
ARID1A	p.Y815*	c.2445T>A	High Grade Glioma	Glioblastoma
ARID1A	p.G2087E	c.6260G>A	Liver Tumors	Hepatoblastoma
ARID1A	p.G2087V	c.6260G>T	Medulloblastoma	Medulloblastoma
ARID1A	p.G277Afs*111	c.828_862delAGGCGGCCCCTCCGCGGCCGGCGGGGGAACTCCCC	Neuroblastoma	Neuroblastoma
ARID1A	p.E2250Rfs*28	c.6746_6747insA	Wilms	Wilms Tumor
ARID1A	p.E1774*	c.5320G>T	Wilms	Wilms Tumor
PIK3CA	p.H1047R	c.3140A>G	Carcinoma	Adenocarcinoma
PIK3CA	p.E542K	c.1624G>A	Carcinoma	Transitional cell carcinoma
PIK3CA	p.E542K	c.1624G>A	Ependymoma	Ependymoma, anaplastic
PIK3CA	p.R38H	c.113G>A	High Grade Glioma	Glioblastoma
PIK3CA	p.E542K, p.R88Q	c.1624G>A, c.263G>A	High Grade Glioma	Astrocytoma anaplastic
PIK3CA	p.R93Q, p.T1025A, p.C378W	c.278G>A, c.3073A>G, c.1134T>G	High Grade Glioma	Glioblastoma
PIK3CA	p.R88Q, p.Y1021H	c.263G>A, c.3061T>C	High Grade Glioma	Glioblastoma
PIK3CA	p.E545K	c.1633G>A	Low Grade Glioma	Astrocytoma, Low Grade
PIK3CA	p.H1047L	c.3140A>T	Medulloblastoma	Medulloblastoma
PIK3CA	p.E542K	c.1624G>A	Medulloblastoma	Medulloblastoma
PIK3CA	p.E545K	c.1633G>A	Medulloblastoma	Medulloblastoma
PIK3CA	p.E542K	c.1624G>A	Non-CNS Germ Cell Tumor	Germinoma
PIK3CA	p.H1047R	c.3140A>G	Other (CNS)	CNS embryonal tumor
PIK3CA	p.E545K	c.1633G>A	Other (Non-CNS)	Epithelioid hemangioendothelioma
PIK3CA	p.E545K	c.1633G>A	Rhabdomyosarcoma	Embryonal rhabdomyosarcoma
PIK3CA	p.C420R	c.1258T>C	Sarcoma (other)	Myxoid liposarcoma
PIK3CA	p.C420R	c.1258T>C	Wilms	Wilms Tumor
PIK3CA	p.N1044Y, p.M1043I	c.3130A>T, c.3129G>T	Wilms	Wilms Tumor

Supplementary Figure 1. OncoPrint showing most common oncogenic alterations in the Extracranial solid tumors (n=512) across the entire analytic cohort

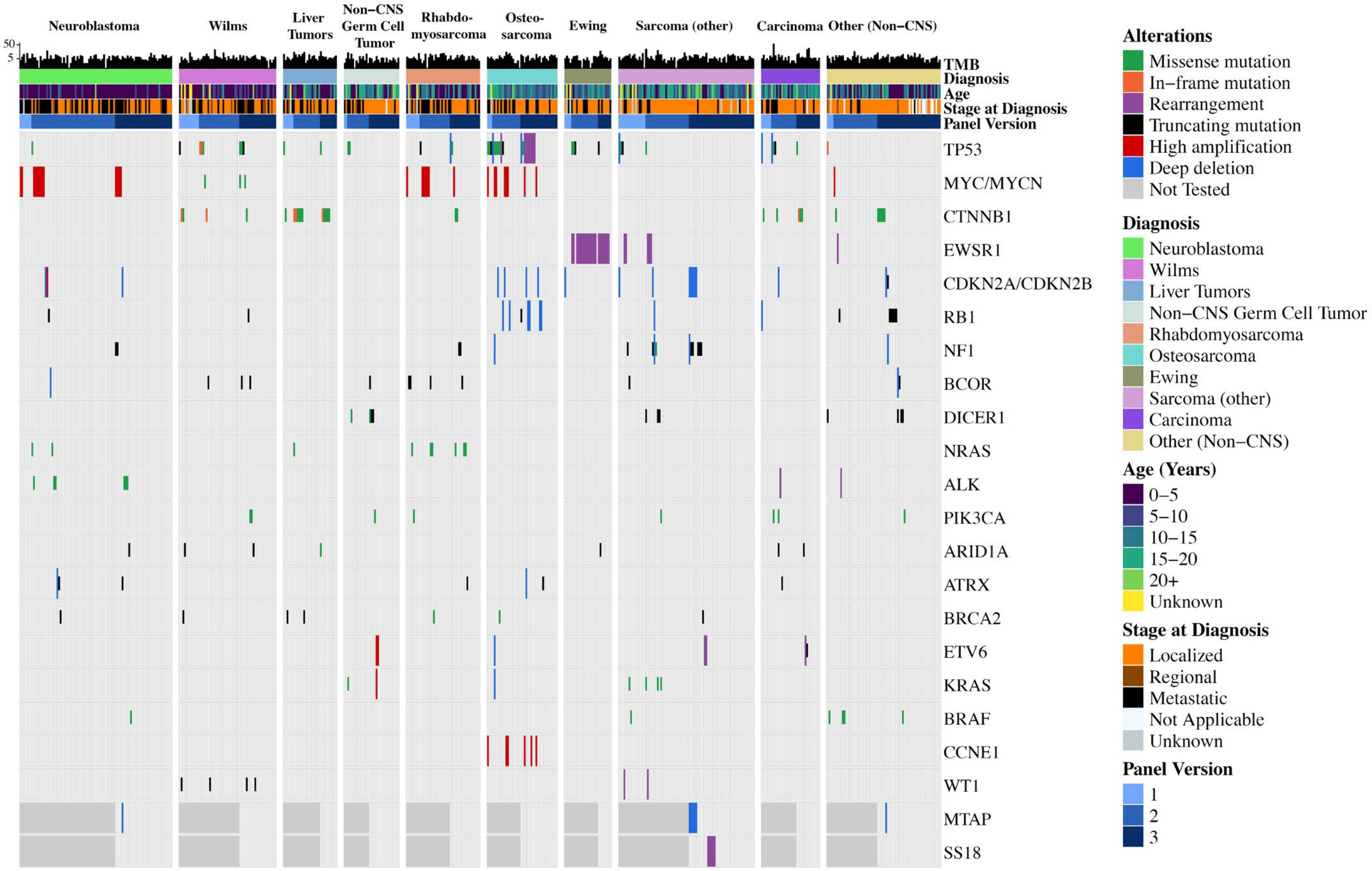

Supplementary Figure 2. OncoPrint showing most common oncogenic alterations in the CNS tumors (n=376) across the entire analytic cohort

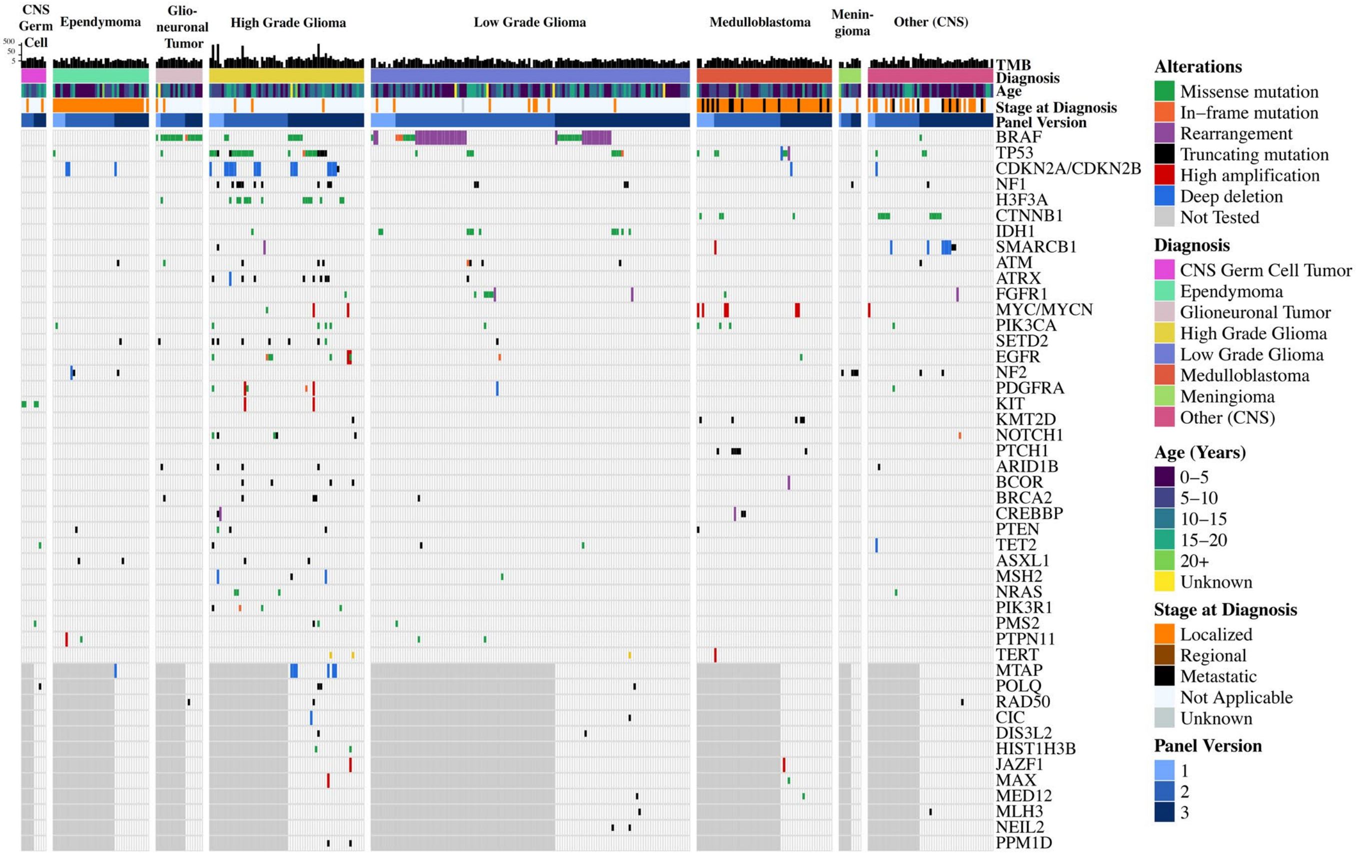

Supplement: Supplementary file 1 — Supplementary Information [file 41467_2024_49944_MOESM1_ESM.pdf]
