# Supplementary material for: Molecular profiling of 888 pediatric tumors informs future precision trials and data-sharing initiatives in pediatric cancer
Source: Nat Commun. 2024 Jul 11;15:5837. doi: 10.1038/s41467-024-49944-0 (PMC11239876; doi:10.1038/s41467-024-49944-0)
Supplement: Supplementary file 3 — Reporting Summary [file 41467_2024_49944_MOESM3_ESM.pdf]

nature portfolio

Corresponding author(s): Suzanne J. Forrest, MD
Katherine A. Janeway, MD

Last updated by author(s): 04/18/2024

Reporting Summary

Nature Portfolio wishes to improve the reproducibility of the work that we publish. This form provides structure for consistency and transparency in reporting. For further information on Nature Portfolio policies, see our Editorial Policies and the Editorial Policy Checklist.

Statistics

For all statistical analyses, confirm that the following items are present in the figure legend, table legend, main text, or Methods section.

n/a	Confirmed	
☐	☒	The exact sample size (*n*) for each experimental group/condition, given as a discrete number and unit of measurement
☐	☒	A statement on whether measurements were taken from distinct samples or whether the same sample was measured repeatedly
☐	☒	The statistical test(s) used AND whether they are one- or two-sided<br>*Only common tests should be described solely by name; describe more complex techniques in the Methods section.*
☒	☐	A description of all covariates tested
☒	☐	A description of any assumptions or corrections, such as tests of normality and adjustment for multiple comparisons
☐	☒	A full description of the statistical parameters including central tendency (e.g. means) or other basic estimates (e.g. regression coefficient) AND variation (e.g. standard deviation) or associated estimates of uncertainty (e.g. confidence intervals)
☒	☐	For null hypothesis testing, the test statistic (e.g. *F*, *t*, *r*) with confidence intervals, effect sizes, degrees of freedom and *P* value noted<br>*Give P values as exact values whenever suitable.*
☒	☐	For Bayesian analysis, information on the choice of priors and Markov chain Monte Carlo settings
☒	☐	For hierarchical and complex designs, identification of the appropriate level for tests and full reporting of outcomes
☒	☐	Estimates of effect sizes (e.g. Cohen's *d*, Pearson's *r*), indicating how they were calculated

Our web collection on statistics for biologists contains articles on many of the points above.

Software and code

Policy information about availability of computer code

Data collection	RedCap (Research Electronic Data Capture)
Data analysis	ClinVar (v. 07_04_2019 release) database<br>gnomAD v2.144 database<br>Memorial Sloan Kettering Precision Oncology Knowledge Base v3.4 (OncoKB)<br>ComplexHeatmap (v. 2.4.3) package

For manuscripts utilizing custom algorithms or software that are central to the research but not yet described in published literature, software must be made available to editors and reviewers. We strongly encourage code deposition in a community repository (e.g. GitHub). See the Nature Portfolio guidelines for submitting code & software for further information.

Data

Policy information about availability of data

All manuscripts must include a data availability statement. This statement should provide the following information, where applicable:

- Accession codes, unique identifiers, or web links for publicly available datasets
- A description of any restrictions on data availability
- For clinical datasets or third party data, please ensure that the statement adheres to our policy

The genomic and clinical datasets generated and analyzed in this study were submitted to the National Cancer Institute's Childhood Cancer Data Initiative (CCDI),

and are available in the database of Genotypes and Phenotypes (dbGaP): Study Accession phs002677.v1.p1 [https://www.ncbi.nlm.nih.gov/projects/gap/cgi-bin/study.cgi?study_id=phs002677.v1.p1]. These data are available under restricted access due to individual privacy concerns, and requests are managed by NCI's Data Access Committee. The comprehensive PRISSMMTM clinical data were shared with the Massachusetts State Cancer Registry who are making it accessible to the National Childhood Cancer Registry (NCCR) and the CCDI. Annotation databases included public resources such as OncoKb, ClinVar, and gnomAD databases. Source data are provided with this paper.

Research involving human participants, their data, or biological material

Policy information about studies with human participants or human data. See also policy information about sex, gender (identity/presentation), and sexual orientation and race, ethnicity and racism.

Reporting on sex and gender	Sex (biologic attribute) is reported for the study population (Table 1) and the source was the electronic medical record.
Reporting on race, ethnicity, or other socially relevant groupings	Race (self-reported) and ethnicity (self-reported) is reported for the study population (Table 1) and the source was the electronic medical record (administrative data).
Population characteristics	Cancer diagnoses were classified as extracranial or intracranial solid tumors, and further sub-classified into disease groupings per Supplementary Table 2.
Recruitment	All patients seen at Dana-Farber/Boston Children's Hospital Cancer and Blood Disorders Center with a suspected or confirmed cancer were eligible to participate in the Profile Cancer Research Study starting in 2013. There was no age limit, and patients were considered pediatric if they were seen by a pediatric provider. Between 2013 and 2019 all pediatric patients with a brain tumor or extracranial solid tumor were offered the opportunity to enroll in the study.
Ethics oversight	This study complies with all relevant ethical regulations and was approved by the Dana-Farber Cancer Institute (DFCI) Institutional Review Board (IRB).

Note that full information on the approval of the study protocol must also be provided in the manuscript.

Field-specific reporting

Please select the one below that is the best fit for your research. If you are not sure, read the appropriate sections before making your selection.

☒ Life sciences ☐ Behavioural & social sciences ☐ Ecological, evolutionary & environmental sciences

For a reference copy of the document with all sections, see nature.com/documents/nr-reporting-summary-flat.pdf

Life sciences study design

All studies must disclose on these points even when the disclosure is negative.

Sample size	This study analyzed a cohort of 888 pediatric cancer patients enrolled on a clinical sequencing study who had successful tumor sequencing between September, 2013 and March, 2019. There was no sample size determination as this is a retrospective cohort study.
Data exclusions	No data were excluded from the analysis.
Replication	This is a retrospective cohort study where replication is not applicable.
Randomization	This is a retrospective cohort study where randomization is not applicable.
Blinding	Blinding was not relevant because this is a retrospective cohort study.

Reporting for specific materials, systems and methods

We require information from authors about some types of materials, experimental systems and methods used in many studies. Here, indicate whether each material, system or method listed is relevant to your study. If you are not sure if a list item applies to your research, read the appropriate section before selecting a response.

Materials & experimental systems

n/a	Involved in the study
☒	☐ Antibodies
☒	☐ Eukaryotic cell lines
☒	☐ Palaeontology and archaeology
☒	☐ Animals and other organisms
☐	☒ Clinical data
☒	☐ Dual use research of concern
☒	☐ Plants

Methods

n/a	Involved in the study
☒	☐ ChIP-seq
☒	☐ Flow cytometry
☒	☐ MRI-based neuroimaging

Clinical data

Policy information about clinical studies

All manuscripts should comply with the ICMJE guidelines for publication of clinical research and a completed CONSORT checklist must be included with all submissions.

Clinical trial registration

Not applicable as this was not an Interventional study.

Study protocol

The study protocol can be submitted as Supplemental Data

Data collection

Pediatric patients consented to the Profile Study between September 2013 and March 2019 with a solid tumor and successful tumor sequencing were included in the study analysis. Tumor samples were requested from the pathology department following patient consent after standard pathology evaluation was complete. Tumor sample acquisition procedures were not altered for these studies, and these clinical samples were most often leftover FFPE (formalin fixed paraffin embedded) specimens in the pathology department. Several additional patients who underwent targeted NGS sequencing with OncoPanel on a similar multi-institution sequencing study and a small number of tumor samples sequenced under a waiver of consent were also included if they had a spindle or round cell sarcoma. The medical records of all the patients with successful OncoPanel sequencing were reviewed to determine clinical and demographic characteristics including sex, race (self-reported), ethnicity (self-reported), pathologic diagnosis, age at diagnosis, and disease stage at diagnosis. Characteristics for the specimen that underwent sequencing were also extracted including timing of sample acquisition including relationship to treatment, and site of tumor (primary site vs. metastatic). Diagnosis was classified according to the International Classification of Diseases for Oncology, version 3.2 (ICD-O-3.2). Tumors were sequenced using the targeted next-generation sequencing OncoPanel platform. Sequencing was performed at the Center for Advanced Molecular Diagnostics (CAMD), a CLIA-certified clinical laboratory in the Department of Pathology at Brigham and Women's Hospital in Boston, Massachusetts.

Outcomes

For analyses of genomic variants, variant call files generated at the time of reporting were utilized. Additional filtering was applied to the existing pipeline output removing mutations found in either the ClinVar (v. 07_04_2019 release)43 or gnomAD v2.144 databases. Tumor mutational burden was calculated by dividing the total remaining number of SNVs or small insertion and deletions (indels) by the total panel size for each version. SNVs and indels were classified as oncogenic if they were labeled as "Oncogenic", "Likely Oncogenic", or "Predicted Oncogenic" per the Memorial Sloan Kettering Precision Oncology Knowledge Base v3.4 (OncoKB). In addition, limited in-house curation was performed (YL, HG, SJF). Specifically, the following variants were further assessed for oncogenicity: 1) loss-of-function (LoF) mutations in tumor suppressor genes (TSG); 2) SNVs and Indels in genes on actionable mutation lists classified as variants of uncertain significance (VUS) and; 3) all fusions involving genes on the actionable mutation lists. OncoPrints were created using the ComplexHeatmap (v. 2.4.3) package.

Genomic alterations were analyzed and matched to the actionable mutation lists (aMOI) of three precision oncology medicine basket clinical trials investigating targeted therapy directed by tumor profiling: NCI-Children's Oncology Group (COG) Pediatric Molecular Analysis for Therapy Choice (MATCH) Screening Trial (NCT03155620), NCI-MATCH Screening Trial (NCT02465060), and ASCO Targeted Agent and Profiling Utilization Registry (TAPUR) Study, Version 3 (NCT02693535). Specific genomic alterations in the participant tumor samples were considered as matches using the following rules: (1) Precise match on either MATCH trial aMOI list (same CNA, SNV, Indel, or LoF mutation for tumor suppressors) taking into account resistance mutations; or (2) Same gene and variant type (eg. activating fusion, amplification or oncogenic SNV/Indel in an oncogene and LoF mutation or deletion in a TSG). For patients with a tumor variant matching a basket trial treatment arm the medical record was reviewed in order to determine whether the patient received a molecularly targeted therapy in the same drug class as the basket trial treatment arm. For patients who received molecularly targeted therapy, the mechanism of obtaining treatment (on a clinical trial, via single patient research protocol, or prescribed off-study) was assessed.

Plants

Seed stocks

Report on the source of all seed stocks or other plant material used. If applicable, state the seed stock centre and catalogue number. If plant specimens were collected from the field, describe the collection location, date and sampling procedures.

Novel plant genotypes

Describe the methods by which all novel plant genotypes were produced. This includes those generated by transgenic approaches, gene editing, chemical/radiation-based mutagenesis and hybridization. For transgenic lines, describe the transformation method, the number of independent lines analyzed and the generation upon which experiments were performed. For gene-edited lines, describe the editor used, the endogenous sequence targeted for editing, the targeting guide RNA sequence (if applicable) and how the editor was applied.

Authentication

Describe any authentication procedures for each seed stock used or novel genotype generated. Describe any experiments used to assess the effect of a mutation and, where applicable, how potential secondary effects (e.g. second site T-DNA insertions, mosiacism, off-target gene editing) were examined.
