# Supplementary material for: Molecular profiling of 888 pediatric tumors informs future precision trials and data-sharing initiatives in pediatric cancer
Source: Nat Commun. 2024 Jul 11;15:5837. doi: 10.1038/s41467-024-49944-0 (PMC11239876; doi:10.1038/s41467-024-49944-0)
Supplement: Supplementary file 4 — Description of Additional Supplementary Files [file 41467_2024_49944_MOESM4_ESM.pdf]

Description of Additional Supplementary Files

File Name: Supplementary Data 1

Description: Gene Targets Included from Three Precision Oncology Trials

File Name: Supplementary Data 2

Description: Number of Patients with each ICD-O Code Categorized into Disease Group and Sub-Group

File Name: Supplementary Data 3

Description: Summary of Genes Covered by Three Versions of OncoPanel
